# Structural brain lesions and action observation therapy outcomes in unilateral cerebral palsy: an exploratory study

**DOI:** 10.3389/fnsys.2026.1766684

**Published:** 2026-05-22

**Authors:** Francy Cruz-Sanabria, Elena Beani, Valentina Menici, Silvia Filogna, Paolo Bosco, Simona Fiori, Laura Biagi, Giuseppina Sgandurra

**Affiliations:** 1 Department of Developmental Neuroscience, IRCCS Fondazione Stella Maris, Pisa, Italy; 2 Department of Clinical and Experimental Medicine, University of Pisa, Pisa, Italy; 3 Laboratory of Medical Physics and Magnetic Resonance, IRCCS Fondazione Stella Maris, Pisa, Italy; 4 Department of Neuroscience and Human Genetics, Meyer Children’s Hospital IRCCS - University of Florence, Florence, Italy

**Keywords:** action observation therapy (AOT), motor function recovery, semi-quantitative MRI (sqMRI), structural MRI biomarkers, unilateral cerebral palsy (UCP)

## Abstract

**Introduction:**

Unilateral cerebral palsy (UCP) results from early brain injury, leading to motor impairments primarily affecting one upper limb. Action Observation Therapy (AOT), which engages the mirror neuron system to enhance motor function, represents a promising rehabilitative approach. However, its efficacy in UCP has been inconsistent across studies, possibly due to methodological differences or individual variability in brain damage. This observational longitudinal study within an intervention group aimed to explore whether structural brain lesions are associated with changes in motor dexterity and bimanual performance following AOT in children with UCP.

**Methods:**

Sixteen children with UCP underwent a structured AOT home training program. Motor function was assessed through the Box and Block Test (BBT) and the Assisting Hand Assessment (AHA) at four or five time points (T0, T1/T1_plus, T2, T3), totaling 64 evaluation sessions. Brain lesions were evaluated using the semi-quantitative magnetic resonance imaging (sqMRI) scale. Data were analyzed using linear mixed-effects models.

**Results:**

A significant increase in BBT scores for the dominant hand (BBT_dom) was observed at all post-treatment time points, while significant improvements in the non-dominant hand (BBT_non_dom) were observed at T2. AHA scores significantly increased at T1 and T2. Lesions involving the temporal lobe, thalamus, brainstem, and corpus callosum showed the strongest associations with reduced improvements in AHA and BBT_non_dom scores.

**Discussion:**

These findings suggest that structural brain lesions may influence the response to AOT in children with UCP. Further studies are required to confirm these results and to evaluate whether tailoring interventions according to neuroanatomical profiles could enhance rehabilitation outcomes in children with UCP.

## 1 Introduction

Cerebral palsy (CP) refers to a group of non-progressive disorders that impact movement and posture due to disturbances in early brain development (Bax et al., 2005). Unilateral spastic cerebral palsy (UCP), a common subtype, arises from congenital or early-acquired brain injury mainly affecting one hemisphere, leading to spasticity predominantly in the contralateral upper limb. Rehabilitation of motor functions is central to the therapeutic approach for UCP. It may involve interventions through intramuscular botulinum toxin A, upper-limb training, physical therapy, special devices, occupational therapy, and action observation training (AOT) (Buccino et al., 2012; Novak et al., 2020; Sakzewski et al., 2009). AOT uses systematic observation of meaningful actions followed by their execution to accelerate functional recovery in patients with motor impairments (Buccino et al., 2006). The theoretical foundation of AOT lies in the activation of the same neural structures during action observation as during the execution of those actions, a phenomenon derived from the “mirror neuron system” (Rizzolatti and Craighero, 2004). Imaging studies have shown that this system is crucial for action understanding and intention coding, imitation, and motor learning (Hari and Kujala, 2009), making it a promising target for therapeutic interventions in UCP.

Despite the promising theoretical underpinnings of AOT, empirical findings regarding its efficacy in improving motor functions in children with CP have been inconsistent (Buchignani et al., 2019). Several studies have reported significant benefits of AOT in enhancing motor skills in CP (Buccino et al., 2012; Jeong and Lee, 2020; Kim et al., 2014; Nuara et al., 2019; Quadrelli et al., 2019; Sgandurra et al., 2013; Simon-Martinez et al., 2025; Simon-Martinez et al., 2018). Indeed, a systematic review by Alamer and colleagues concluded that AOT is a promising intervention for upper limb rehabilitation in children with hemiplegic CP (Alamer et al., 2020). However, other studies have failed to confirm these findings, with some authors reporting no significant improvements (Kim, 2020; Kirkpatrick et al., 2016). Moreover, a meta-analysis by Abdelhaleem and colleagues (Abdelhaleem et al., 2021) found insufficient evidence to draw firm conclusions about the effectiveness of AOT in CP rehabilitation. These discrepancies could stem from methodological differences between studies or from clinical heterogeneity among treated patients, highlighting the need for identifying markers that could predict a patient’s response to AOT.

Among the markers that could predict AOT response, we can include neuroanatomical features. Several studies have demonstrated the potential of AOT to induce functional reorganization within motor-related brain areas, leading to improved motor outcomes in children with CP. The neural mechanisms underlying motor function, particularly the action observation network (AON) and the sensorimotor network (SMN) have been extensively studied in UCP. In a previous study conducted by our group, exploring the reorganization of AON and SMN in children with UCP using functional magnetic resonance imaging (fMRI), findings revealed that children with UCP showed higher lateralization of AON compared to typically developing (TD) peers, which was negatively correlated with clinical performance (Sgandurra et al., 2018). Consistently, in a pilot study performed by our group investigating the impact of AOT on brain reorganization in children with congenital hemiplegia using fMRI, we found that after three weeks of AOT, the experimental group showed a shift toward a more bilateral representation of the AON, suggesting that bilateral AON activation could be related with better motor performance in UCP. Moreover, the experimental group demonstrated significantly higher activation in the SMN compared to the control group, particularly in regions such as the ipsilesional primary motor area, pre-central gyrus, and supplementary motor area (Sgandurra et al., 2020). In the same line, the group of Buccino and colleagues (Buccino et al., 2018) found that children who underwent AOT exhibited significant improvements in motor function accompanied by stronger activation in the parietal-premotor circuit, particularly in the left premotor cortex, inferior frontal gyrus, and superior temporal gyrus.

Despite these insights, there is a notable gap in the literature regarding structural MRI characteristics that could predict the efficacy of AOT in children with UCP. Recently, Beani et al. (2024) observed moderate to strong associations between upper limb functional impairment and structural brain characteristics, as measured through a semiquantitative MRI scale (sqMRI) (Fiori et al., 2014). Specifically, associations between sqMRI scores contralateral to the more affected side and upper limb functional impairment, as measured through the Manual Ability Classification System (MACS) and the Box and Block Test (BBT), were identified. More severe brain injuries significantly correlated with poorer function in the non-dominant hand (Beani et al., 2024). These findings suggest that structural MRI markers could serve as potential predictors of response to rehabilitation strategies like AOT. The present study aims to explore whether structural brain lesions are associated with changes in motor dexterity and bimanual performance following AOT in children with UCP. This study could enable the identification of MRI markers that may be associated with the effect of AOT on motor function.

## 2 Materials and methods

### 2.1 Participants

Children with UCP enrolled in the Tele-UPCAT project (Beani et al., 2023; Sgandurra et al., 2021) were selected for this study. Inclusion criteria were: (1) children, adolescents, or young adults aged 5 to 20 years; (2) confirmed having a diagnosis of UCP; (3) cognitive level within normal limits (IQ > 70); (4) availability to commit to a home program of intensive therapy for 3 weeks. Further inclusion criteria were the availability of a complete brain MRI exam to classify brain lesions according to different scales. Exclusion criteria were (1) orthopedic surgery or intramuscular botulinum toxin A injection in the upper limb prior to the starting of the rehabilitation session.

The upper limb function was assessed using specific outcome measures according to the study protocol. Specifically, the Box and Block Test (BBT) (Mathiowetz et al., 1985) measures unimanual dexterity in the activity domain, assessing both dominant (less affected) and non-dominant (more affected) hand separately. The Assisting Hand Assessment (AHA) (Krumlinde-sundholm et al., 2003) measures upper limb function during bimanual activities by evaluating spontaneous use of the assisting hand during a semistructured age-appropriated session.

The trial was approved by the Tuscan Pediatric Ethics Committee (number 169/2016) and registered at: http://www.clinicaltrials.gov (NCT03094455) on 16 March 2017. All parents provided written informed consent to participate in the trial.

### 2.2 Instruments

#### 2.2.1 Clinical hand function assessment

##### 2.2.1.1 Gross manual dexterity assessment

The Box and Block Test (BBT) is a standardized test used to measure gross manual dexterity, widely used with adults and later adopted also in pediatric age (Mathiowetz et al., 1985). It is quick, easy to administer, and cost-effective. The test apparatus consists of a box divided into two compartments by a central partition and 150 wooden blocks, each measuring 25 mm. Participants are instructed to move the blocks from one compartment to the other as quickly as possible within a one-minute timeframe. The number of blocks successfully moved is recorded, with a higher count indicating better manual dexterity. In the UCP cohort, the test is first performed with the dominant hand, followed by the non-dominant hand. A difference of 7 blocks was considered an indicator of clinically important change (Liang et al., 2021).

##### 2.2.1.2 Assisting hand assessment

The Assisting Hand Assessment (AHA) is designed to evaluate how effectively children with unilateral upper limb dysfunction, such as obstetric brachial plexus palsy or UCP, use their non dominant hand during a semi-structured play session (Krumlinde-Sundholm et al., 2007). It is validated for children from 18 months up to 18 years (Louwers et al., 2016), with age-appropriated comparable sessions (from free play to different board games) in which objects requiring bimanual use are presented. After videotaping the session, the score is made by a certified scorer; the latest version, the 5.0, is composed of 20 items each scored with 4 points scale, which are processed based on Rasch analysis, returning a total sum score (1–80) converted also in AHA units score (1–100). Higher scores mean more symmetric hand use and better non-dominant side abilities. A difference of 5 AHA units is considered a clinically relevant change beyond measurement error.

#### 2.2.2 Neuroimaging assessment

MRI data of all participants were acquired at the IRCCS Stella Maris Foundation, by using either a 1.5 T or 3 T MRI scanner (Signa Horizon 1.5 T, Signa Premier 3 T, GE Healthcare, Milwaukee, WI). The standard clinical protocol included two-dimensional T1-weighted, T2-weighted, and T2*-weighted- sequences, as well as three-dimensional T1-weighted, T2w FLAIR, and T2* Susceptibility weighted (SWI) sequences. The MR images were retrospectively evaluated using a reliable and validated semi-quantitative scoring system (Fiori et al., 2014; Fiori et al., 2015). An experienced pediatric neurologist (SF) assessed the images.

##### 2.2.2.1 Brain lesion MRI assessment

Brain lesions on clinical MRI images were evaluated using a validated semi-quantitative (sqMRI) scoring system (Fiori et al., 2014). This method is based on a six-axial-slice template, with anatomical regions identified using specific MRI slices. The assessment systematically evaluates both hemispheric and subcortical structures on each hemisphere (right and left), generating subscores for the frontal, parietal, temporal, and occipital lobes; for subcortical structures including caudate, lenticular nuclei (putamen and globus pallidus), posterior limb of the internal capsule (PLIC), thalamus, and brainstem; as well as for the cerebellum and corpus callosum. Higher scores indicate greater involvement of the respective brain regions. Brain MRI images were also classified using the Surveillance of Cerebral Palsy in Europe (SCPE) classification system, based on the Magnetic Resonance Imaging Classification System (MRICS) (Himmelmann et al., 2017). The MRICS categorizes findings into five main groups: maldevelopments (Type I), predominant white matter injury (Type II), predominant gray matter injury (Type III), miscellaneous (Type IV), alongside a category for normal findings (Type V).

### 2.3 Procedure

Tele-UPCAT study was designed as an intention-to-treat clinical trial and implemented as a randomized AOT rehabilitation program and a Standard Care (SC) group, allocation-concealed, waitlist-controlled, and evaluator-blinded investigation. Participants enrolled after baseline evaluations, were randomized into either the immediate AOT or SC group. Participants underwent assessments at baseline (T0) and after a 3-week initial intervention period, which involved AOT for the experimental group and SC for the control group (T1). The SC group received typical care and after completing the SC phase, this group transitioned to the AOT intervention, followed by a secondary assessment upon completion (T1 plus). Both groups were subsequently evaluated at 8 weeks (T2) and 24 weeks (T3) post-intervention.

The intervention phase consisted of 15-day cycles of goal-oriented activities with increasing complexity over the training period (each daily session lasted approximately 1 h: alternating observation and action phases). The program began with eight unimanual training sessions, followed by seven bimanual sessions. The training was delivered through the Tele-UPCAT system (provided directly to the participant’s home), which included an all-in-one computer equipped with specialized software, a toy kit, and Actigraph devices.

During the Standard Care (SC) phase, participants continued their usual care without receiving any additional training. The Italian National Health System provides at least one or two therapy sessions per week, with a total duration of approximately 1 h a week, and this corresponded to the therapy received by all participants in our trial, as confirmed by the diaries completed by all families throughout the study. In contrast, the AOT intervention, as an intensive and structured home-based program delivered over 3 consecutive weeks (5 days per week, approximately 1 h per day), resulted in a substantially higher treatment intensity compared to SC.

Clinical assessments were conducted under controlled conditions by trained therapists, even when performed in the home setting. All outcome measures, including the Assisting Hand Assessment (AHA) and Box and Block Test (BBT), were video-recorded using standardized procedures. Subsequently, scoring was performed by certified assessors who were blinded to both group allocation and assessment time point. In particular, AHA evaluations were conducted by certified raters following standardized administration and scoring protocols, ensuring high reliability. The use of video recordings allowed for detailed and repeated evaluation, further enhancing scoring accuracy and consistency across time points.

### 2.4 Data analysis

To evaluate the effects of Action Observation Therapy (AOT) on motor dexterity and to explore structural MRI predictors of treatment response, we employed linear mixed-effects models (LMMs). We modeled the scores obtained from the BBT for both the dominant (BBT_dom) and non-dominant (BBT_non_dom) hands and the AHA scores as dependent variables. The models incorporated a random effect for each participant (denoted by “Code”) to capture individual variability. The fixed effects included time points (TIME: T0, T1/T1plus, T2, T3), Age, and Sex, enabling us to assess changes in motor dexterity throughout AOT while adjusting for potentially confounding factors. Indeed, several factors may impact recovery following early brain insult, including injury features (extent, severity, and location), age at the time of insult, and sex (Anderson et al., 2011). Moreover, the broad age range may introduce developmental variability; to account for this, age was included as a covariate in all mixed-effects models. Three primary models were developed: Model 1.1 with BBT_dom as the dependent variable, Model 1.2 with BBT_non_dom as the dependent variable, and Model 1.3 with AHA scores as the dependent variable.

To explore the contribution of structural MRI features to motor function over time, we developed secondary linear mixed-effects models by incorporating semi-quantitative MRI (sqMRI) scores as additional fixed effects. SqMRI variables were entered separately into each model and included scores for the frontal, temporal, parietal, and occipital lobes, as well as the caudate nucleus, lenticular nuclei, posterior limb of the internal capsule (PLIC), thalamus, and brainstem—classified as either ipsilesional or contralesional. Additional regions analyzed included the corpus callosum and cerebellum. All secondary models included TIME, age, sex, and lesion hemisphere (right vs. left) as fixed effects, and participant ID as a random effect. Moreover, as testing multiple brain regions increases the risk of type I error, *p*-values were adjusted using the false discovery rate (FDR) method, with statistical significance set at *p* < 0.05.

Given the high collinearity between sqMRI variables and lesion classification under the MRICS, the effect of MRICS was examined in separate mixed-effects models. Specifically, three models (one per outcome: BBT_dom, BBT_non_dom, and AHA) were fitted with MRICS classification (Type III vs. Type II) as a fixed effect, adjusting for TIME, age, and sex. MRICS Types I, IV, and V were excluded from analysis due to low representation in the sample. All analyses were conducted using R version 4.4.1.

## 3 Results

**Figure 1:**
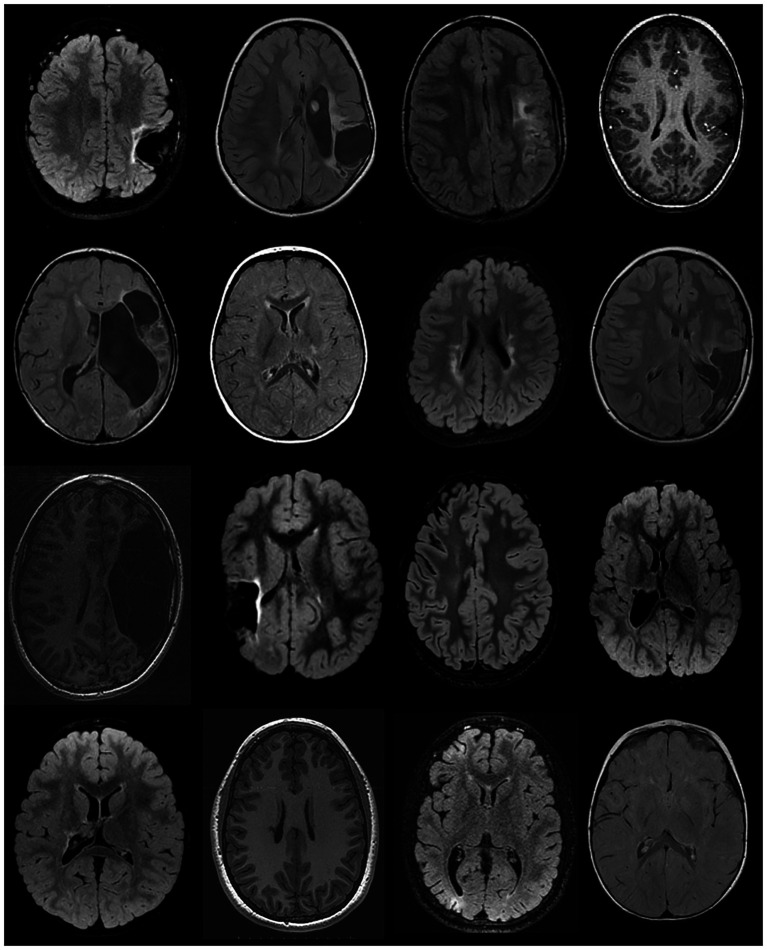
Representative examples of structural brain lesions in children with unilateral cerebral palsy, displayed on FLAIR and T1-weighted magnetic resonance images. Images illustrate variability in lesion location and extent across participants. The left hemisphere is shown on the right side of each image according to radiological convention.

The study included 16 children with UCP assessed at time points (T0, T1, T1_plus T2, T3), totaling 64 evaluation sessions. Demographic and clinical characteristics are presented in Table 1 and Figure 1. Significant differences between patients with predominant Right vs. Left lesions were found in baseline AHA scores. The involvement of each brain region, as measured by sqMRI scores observed in the overall sample are summarized in the Supplementary Table S1.

**Table 1:** Clinical data.

Variable	All sample (*n =* 16)	Right hemiplegia, left hemisphere lesion (*n =* 9)	Left hemiplegia, right_hemisphere lesion (*n =* 7)	*p*-value
Age, (median [IQR])	9.41 [8.55, 11.49]	9.59 [9.04, 10.73]	8.57 [7.04, 11.54]	0.223
Sex, male (%)	8 (50)	5 (55.6)	3 (42.9)	1
Baseline AHA (median [IQR])	48.50 [40.75, 58.50]	41.00 [39.00, 55.00]	53.00 [48.50, 66.00]	0.039*
Baseline BBT dominant (median [IQR])	52.50 [46.75, 59.00]	53.00 [48.00, 62.00]	51.00 [46.50, 58.00]	0.672
Baseline BBT non-dominant (median [IQR])	17.00 [12.75, 34.00]	13.00 [11.00, 33.00]	17.00 [16.00, 36.50]	0.203
GMFCS, *N* (%)				0.294
GMFCS_I	13 (81.2)		7 (100)	
GMFCS_II	3 (18.8)	3 (33.3)	0	
GMFCS_III	0 (0.0)	0 (0.0)	0 (0.0)	
GMFCS_IV	0 (0.0)	0 (0.0)	0 (0.0)	
MACS, *N* (%)				0.176
MACS_I	4 (25.0)	1 (11.1)	3 (42.9)	
MACS_II	6 (37.5)	3 (33.3)	3 (42.9)	
MACS_III	6 (37.5)	5 (55.6)	1 (14.3)	
MACS_IV	0 (0.0)			
MRICS, *N* (%)				0.504
Type I	1 (6.2)	1 (11.1)	0 (0.0)	
Type II	5 (31.2)	2 (22.2)	3 (42.9)	
Type III	10 (62.5)	6 (66.7)	4 (57.1)	
Type IV	0 (0.0)	0 (0.0)	0 (0.0)	
Type V	0 (0.0)	0 (0.0)	0 (0.0)	

GMFCS, Gross Motor Function Classification System; MACS, Manual Ability Classification System; MRICS, MRI classification system. (*) Significant *p*-value (<0.05) of non-parametric comparisons between groups (Left vs. Right hemiplegia).

**Figure 2:**
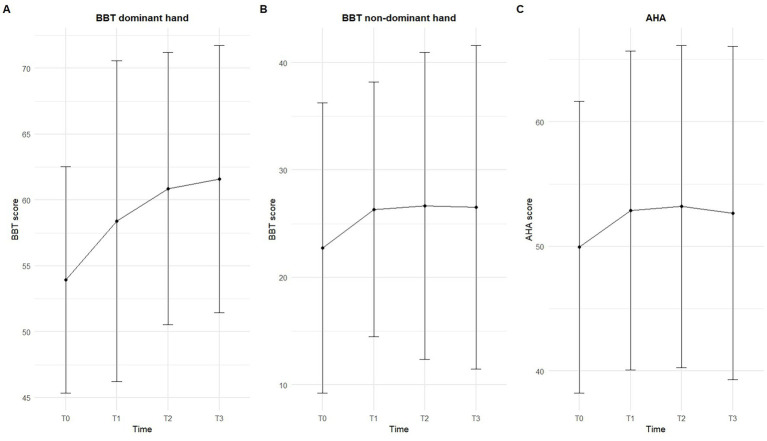
Longitudinal changes in motor function outcomes across time points. Mean scores and standard deviations are shown for **(A)** Box and Block Test (BBT) of the dominant hand, **(B)** BBT of the non-dominant hand, and **(C)** Assisting Hand Assessment (AHA). T0: Baseline; T1: post-intervention; T2: 8 week follow-up; T3: 24 week follow-up. Error bars represent standard deviations.

AHA and BBT scores at T0, T1, T2, and T3 are summarized in Figures 2A–C. Primary linear mixed-effects models indicate that BBT_dom scores significantly improved at T1, T2, and T3 compared to T0; moreover, age had a significant positive effect, with each additional year of age associated with a score increase, while sex was not significant (Model 1.1). BBT_non_dom scores showed a significant increase at T2, with no significant effects of Age or Sex (Model 1.2). AHA scores increased significantly over time. Specifically, scores at T1 and T2 were significantly higher than at T0, while age and sex were not significantly associated with AHA scores (Model 1.3) (see Table 2).

**Table 2:** Primary linear mixed-effect models examining changes in clinical outcomes over time.

Model	Variable	Coefficient	Std_error	*T*-value	*P*-value
Model 1. 1 Dependent variable: BBT dominant hand scores	(Intercept)	29.652	7.063	4.198	0.001
	TIMET1	4.1	1.633	2.511	0.016*
	TIMET2	5.619	1.644	3.418	0.001**
	TIMET3	5.316	1.698	3.131	0.003**
	Age	2.13	0.593	3.593	0.002**
	Sex (Male)	4.207	4.111	1.023	0.325
Model 1.2 Dependent variable: BBT non-dominant hand scores	(Intercept)	10.638	8.832	1.205	0.238
	TIMET1	1.914	1.055	1.814	0.077
	TIMET2	2.886	1.075	2.686	0.010*
	TIMET3	2.238	1.186	1.888	0.065
	Age	1.057	0.702	1.507	0.139
	Sex (Male)	2.205	6.469	0.341	0.739
Model 1.3. Dependent variable: AHA scores	(Intercept)	44.518	7.889	5.643	<0.001
	TIMET1	2.895	0.814	3.559	0.001**
	TIMET2	2.329	0.848	2.745	0.009**
	TIMET3	1.589	0.952	1.669	0.101
	Age	0.424	0.604	0.703	0.485
	Sex (Male)	2.001	6.328	0.316	0.757

Separate models were run for each outcome variable: Assisting Hand Assessment (AHA) and Box and Block Test for non-dominant and dominant hand. All models were adjusted for time (T1, T2, T3), age, sex, and lesioned hemisphere (right vs. left). Asterisks indicate statistical significance (*p < 0.05, **p < 0.01).

Secondary models showed that the strongest associations emerged between motor outcomes (BBT_non_dom and AHA changes over time), and ipsilesional temporal, thalamus, brainstem, and corpus callosum (adjusted *p*-value < 0.01**). Additional associations were also observed with frontal and occipital lobe lesions (*p* < 0.05*). Instead, no region was associated with changes in the BBT_dominant. These results indicate that greater lesion involvement in these regions, as reflected by higher sqMRI scores, was associated with reduced response in the non-dominant hand to the AOT treatment over time. Age was positively associated with motor outcomes, while sex did not significantly influence outcomes. Table 3 reports coefficients, *p*-values, and FDR-corrected p-values for each brain region evaluated. Finally, models examining the effects of lesion type and timing (MRICS categories) on motor outcomes (BBT_dom, BBT_non_dom, and AHA) adjusted for TIME, Age, and Sex revealed no significant effect of MRICS variables (see Supplementary Table S2).

**Table 3:** Secondary linear mixed-effects models examining association between structural brain lesions and clinical outcomes.

Predictor	AHA		BBT_non dominant		BBT dominant	
	Estimate	Adjusted *p*-value	Estimate	Adjusted *p*-value	Estimate	Adjusted *p*-value
Ipsilesional frontal	−8.751	0.071	−7.835	0.041*	−6.406	0.116
Ipsilesional temporal	−7.942	0.008**	−7.156	0.006**	−1.553	0.711
Ipsilesional parietal	−3.247	0.396	−4.169	0.196	−2.687	0.668
Ipsilesiona occipital	−7.891	0.031*	−6.734	0.023*	−2.103	0.668
Ipsilesional_PLIC	−13.088	0.198	−12.541	0.196	8.144	0.668
Ipsilesional_Lenticular	−11.672	0.155	−8.427	0.196	−9.809	0.183
Ipsilesional_Thalamus	−19.047	0.008**	−16.219	0.007**	−1.295	0.975
Ipsilesional_Brainstem	−19.047	0.008**	−16.219	0.007**	−1.295	0.975
Corpus callosum	−12.148	0.008**	−9.178	0.013*	−6.261	0.116
Contralesional frontal	19.160	0.155	14.128	0.196	0.444	0.975
Contralesional temporal	19.160	0.155	14.128	0.196	0.444	0.975
Contralesional parietal	9.454	0.155	6.797	0.196	−0.123	0.975
Contralesiona occipital	19.160	0.155	14.128	0.196	0.444	0.975
Cerebellum	−9.839	0.444	−6.630	0.536	−13.588	0.282

Separate models were run for each brain region (sqMRI score) and each outcome variable: Assisting Hand Assessment (AHA) and Box and Block Test for the dominant and non-dominant hands. All models were adjusted for time (T1, T2, T3), age, sex, and lesioned hemisphere (right vs. left). *P*-values were corrected for multiple comparisons using the False Discovery Rate (FDR) method. Asterisks indicate statistical significance after FDR correction (*p < 0.05, **p < 0.01). Regions with no observed lesions across the sample (Ipsilesional and Contralesional Caudate, Contralesional PLIC, Contralesional Lenticular nucleus, Contralesional Thalamus, and Contralesional Brainstem) were excluded from modeling.

## 4 Discussion

To our knowledge, the current study is the first to explore structural brain lesions potentially associated with AOT effects in the context of rehabilitation interventions in children with UCP. Our results demonstrate a significant increase in BBT scores for the dominant hand at all post-treatment time points, for the non-dominant hand at T2, and for the AHA scores at T1 and T2. These results may be related to preexisting disparities in motor function between the two hands. Children with UCP may exhibit greater baseline dexterity in the dominant hand due to asymmetric neural reorganization patterns and compensatory cortical plasticity. The limited but delayed improvement in BBT_non_dom may indicate a slower but possible plastic response in the less functional hemisphere. The observed improvements in AHA scores at T1 and T2 suggest early and meaningful enhancements in bimanual coordination during and shortly after the intervention period. The AHA, which evaluates the child’s ability to integrate the non-dominant hand in everyday tasks, captures functional gains that may not be fully reflected in unimanual assessments like the BBT. The early rise in AHA scores may suggest that AOT is associated with improvements in both isolated motor skills and the incorporation of the impaired hand into functional activities.

Moreover, this study examined how a semiquantitative score, developed to describe structural brain damage, may be related to functional improvements in motor tasks after an AOT intervention. Lesion of specific brain areas, particularly the ipsilesional temporal, thalamus, brainstem, and corpus callosum, showed the more significant associations with changes in AHA and BBT non-dominant across time (*p* < 0.01). Other regions showing associations include Ipsilesional Occipital and Ipsilesional Frontal (*p* < 0.05). Instead, no region was associated with changes in the BBT_dominant. This finding suggests that damage to these regions, may be associated with the capacity to adapt or reorganize in response to AOT, potentially contributing to variability in the observed therapeutic response.

Previous studies did not observe significant associations between other structural MRI biomarkers and motor outcomes after intervention (Friel et al., 2014; Islam et al., 2014). Specifically, in the study by Friel et al., where the structural MRI marker considered was corticospinal tract dysgenesis, hand function was assessed pre- and post-treatment using the AHA and Jebsen–Taylor Hand Function Test (JTHFT), and the intervention was based on Hand-Arm Bimanual Intensive Training (HABIT); the authors found that peduncle asymmetry correlated with baseline AHA and JTHFT scores but was not associated with improvements in these measures post-training. Likewise, the interventional study by Islam et al., based on Constraint-Induced Movement Therapy (CIMT), evaluates clinical outcomes through the JTHFT, AHA, and the Melbourne Assessment, and brain lesions with a visual assessment of conventional structural MRI. Although they found significant improvements on the JTHFT and AHA, there was no relationship between training-induced motor function changes and the type, location, or extent of lesions. Other authors have reported mixed results, such as in the study by Schertz et al., where a greater brain injury (measured using a radiological scoring method) was associated with enhanced improvements in bimanual function (AHA) but poorer outcomes in unimanual performance (JTHFT) after an intervention based on HABIT (Schertz et al., 2016).

Several methodological differences may explain these contrasting findings across studies. First, differences in intervention approaches may influence the relationship between brain structure and functional recovery. HABIT, for instance, is based on motor learning principles, including repetitive practice and skill progression, actively engaging participants in bimanual tasks throughout the training period (Friel et al., 2014). Conversely, CIMT focuses on constraining the nonparetic hand while engaging the paretic hand in intensive, repetitive training (Kuhnke et al., 2008). The nature of these interventions may influence whether and how brain structural markers correlate with functional gains. Second, differences in MRI-based lesion assessment methods could also account for the discrepancies observed across studies. In the study by Friel et al., corticospinal tract dysgenesis was measured as peduncle asymmetry on T1-weighted MRI, providing an indirect assessment of the integrity of motor pathways. Meanwhile, Islam et al. relied on conventional structural MRI to visually assess brain lesion characteristics, an approach that may lack quantitative specificity regarding lesion severity and location. Schertz et al. used a quantitative scoring system based on topographical MRI patterns, considering multiple domains, such as the extent of white and gray matter damage and major tract involvement, but without directly linking lesions to specific functional circuits.

In contrast, our study utilized AOT as the intervention and the sqMRI scale as the lesion assessment method (Fiori et al., 2014), which provides a region-specific measure of lesions. Unlike conventional visual MRI assessments or global radiological scores, sqMRI allows for a quantification of lesion presence in specific brain regions, offering a potential advantage in detecting potential associations between lesion localization and motor outcomes. These methodological differences highlight the importance of further exploring neuroimaging biomarkers and intervention paradigms using more homogeneous approaches between studies to confirm potential structural MRI markers associated with functional recovery in neurodevelopmental conditions.

Brain regions that we found as potentially associated with AOT response have been previously associated with motor function in UCP. In dyskinetic CP, reduced thalamic volume and increased lesion severity have been associated with greater motor impairment (Laporta-Hoyos et al., 2014; Sanvido et al., 2022). Similarly, disruption of thalamocortical and corticospinal tracts has been linked to motor dysfunction, underscoring the thalamus’s critical role in both sensory and motor pathways in CP (Caldú et al., 2024). The integrity of the corpus callosum has also been shown to play an important role in upper extremity function in children with UCP due to its role in integrating activity from both hemispheres to optimize bilateral hand function and bimanual skills (Weinstein et al., 2014). Additionally, studies in CP have demonstrated significant correlations between fractional anisotropy values within the corticospinal tracts, posterior corpus callosum, and thalamic radiations with motor dysfunction (Hoon Jr et al., 2009; Lee et al., 2011; Yoshida et al., 2010). Further supporting these findings, the severity of white matter injury in the prefrontal lobe, thalamus, internal capsule, corpus callosum, and brainstem has been shown to negatively correlate with motor function in patients with occult spastic diplegic cerebral palsy (Mu et al., 2014). Although we utilized a semiquantitative, rather than a quantitative, approach to characterize brain lesions, our results align with these prior studies and provide potential neuroimaging markers associated with motor function outcomes in patients with UCP.

Only two studies have previously explored the association between AOT and brain imaging markers in UCP by using functional MRI (fMRI). In the first from our group, authors demonstrated the potential of AOT to promote brain reorganization and improve functional outcomes in children with UCP, with findings suggesting that motor system activation expands over time in response to intervention, showing a shift toward a more bilateral representation of the AON and higher activation in the sensorimotor network (Sgandurra et al., 2020). Similarly, in the second study, children who underwent AOT exhibited significant improvements in motor function accompanied by stronger activation in the parietal-premotor circuit, particularly in the left premotor cortex, inferior frontal gyrus, and superior temporal gyrus (Buccino et al., 2018). However, neither of these studies explores predictors of AOT efficacy based on structural brain characteristics.

The present study advances this field by identifying the brain structural markers that may be associated with motor recovery in children with UCP undergoing AOT. Our findings suggest that damage to key regions, including the thalamus, brainstem, and corpus callosum, may be associated with reduced improvement in motor function, as measured by the BBT and AHA scores. Unlike prior research that primarily described associations between AOT response and brain functional correlates, this study provides novel insights into how specific neuroanatomical regions may be associated with variability in response to targeted therapeutic interventions like AOT. These findings support the notion that a one-size-fits-all rehabilitation approach may not be optimal and underscore the potential value of more individualized intervention strategies that take into account the neuroanatomical variability characteristic of CP. Moreover, the use of a semiquantitative MRI score offers several advantages. Notably, the score can be easily calculated from standard FLAIR images without requiring the acquisition of specific sequences or complex preprocessing steps, making it a practical and accessible tool for clinical and research applications.

Current evidence suggests that AOT may have modest benefits for improving arm and hand function in adult stroke patients. However, the low certainty of evidence results highlights the need for further high-quality research to confirm its efficacy (Borges et al., 2022). In contrast, several studies have demonstrated the efficacy of AOT in children with unilateral or bilateral CP, emphasizing its great potential in pediatric rehabilitation (Buccino et al., 2012; Kim et al., 2014; Sgandurra et al., 2013). Therefore, the effects of AOT may differ between adults with acquired stroke and children with congenital brain lesions, such as unilateral CP. This divergence may stem from distinct mechanisms underlying motor function recovery. In adult stroke, recovery is primarily driven by reorganizing function within the ipsilesional cortex to restore the motor cortex-spinal cord connection (Burzi et al., 2016). Conversely, in congenital brain lesions, neuroplastic processes depend on the phase of brain maturation. During early development, bilateral motor projections from the primary motor areas initially connect each hemisphere to both body sides. These projections typically retract during maturation but may persist in cases of early cerebral damage, resulting in contralesional reorganization of motor function (Eyre, 2007). Therefore, in congenital brain injuries, two types of reorganization can be observed: ipsilesional reorganization within spared cortical tissue of the affected hemisphere and contralesional reorganization in the undamaged cortex (Staudt et al., 2004). Therefore, recovery during early development is influenced by critical periods for neuroplasticity (Anderson et al., 2011; Burzi et al., 2016). Although we analyzed the influence of lesion timing and type, as classified by the MRI-SCPE system, no significant associations with outcomes were identified (Supplementary Table S2). However, it is crucial to note that our sample size did not provide sufficient numerical representation for each MRI-SCPE category, limiting the analysis for this variable.

Based on the rationale of AOT, it can be expected that patients with more preserved regions belonging to the mirror neuron system (MNS) may better benefit from interventions implicating imitation processes. In the present study, the ipsilesional frontal region was associated with therapy effects. Further studies with a larger sample size should confirm these results. In addition to frontal and subcortical regions, our findings also highlight the potential involvement of the temporal lobe in the response to AOT. In particular, the superior temporal cortex has been consistently implicated in the visual analysis of biological motion and action observation. Within the mirror neuron framework, the superior temporal sulcus provides critical perceptual input to the fronto-parietal mirror network, contributing to the encoding of observed actions (Rizzolatti and Craighero, 2004). Therefore, alterations in temporal regions may affect the perceptual processing and interpretation of observed actions, which are essential components of imitation-based learning. Moreover, it is relevant to highlight that the MNS may extend beyond the classical cortical areas, such as the ventral premotor cortex and inferior parietal lobule, to include subcortical regions like the basal ganglia and thalamus, suggesting a broader network supporting MNS processes (Bonini, 2017; Errante et al., 2023; Errante and Fogassi, 2020). Our preliminary results involving subcortical regions open avenues for future research into the relationship between the integrity of other regions besides the classical mirror neuron system and the effectiveness of AOT interventions.

This study is a first step into exploring the potential associations between structural brain markers and motor function improvements following AOT in children with UCP. However, several limitations should be considered. First, the relatively small sample size limits the generalizability of the findings and may reduce the statistical power to detect more subtle effects. Nevertheless, the present study should be considered exploratory and represents an initial step toward generating hypotheses to be tested in larger samples. Moreover, variability in neuroanatomical profiles observed in UCP suggests that a one-size-fits-all rehabilitation approach may not be enough, future studies with larger sample may contribute to identify neuroanatomical patterns that may guide more personalized intervention strategies could better account for the and treatment responses observed in children with UCP. Second, although adherence to the AOT home training was monitored, the study did not include an objective, quantitative evaluation of adherence variability across participants, which could influence outcomes. Nevertheless, in line with the original Tele-UPCAT protocol, all participants completed the full set of training sessions, with no missed sessions or drop-outs reported, suggesting excellent compliance. Furthermore, families maintained daily diaries to monitor routine activities and potential confounding therapies. Another important aspect to consider is the difference in treatment intensity between Standard Care (SC) and the AOT intervention. While SC consisted of usual care, typically involving one to two therapy sessions per week, AOT was delivered as an intensive, structured program over 3 weeks. Therefore, it cannot be excluded that differences in treatment intensity may have contributed, at least in part, to the observed changes. Third, while this study identified specific brain regions showing significant associations with treatment response, the cross-sectional nature of MRI data precludes understanding the longitudinal evolution of neuroplastic changes induced by AOT. Moreover, although the risk of type I error associated with testing multiple brain regions was addressed using the false discovery rate (FDR) method, the possibility of false positive findings cannot be completely excluded, given the exploratory nature of the study. In addition, the broad age range of participants introduces developmental variability that may have influenced the results. Although this was addressed by including age as a covariate in the mixed-effects models, residual heterogeneity cannot be excluded. Furthermore, although a significant baseline difference in AHA scores between lesion hemisphere groups was observed, this was addressed within the linear mixed-effects modeling framework, which captures within-subject changes over time and reduces the potential influence of initial group differences on longitudinal estimates. In addition, lesion hemisphere (right vs. left) was included as a fixed effect in the secondary models, allowing further adjustment for potential group differences in these analyses.

Regarding the choice of MRI assessment method, we employed a semi-quantitative MRI (sqMRI) scale, which represents a clinically feasible and widely applicable approach, particularly in pediatric populations. One of its main advantages is that it can be derived from conventional MRI sequences (standard FLAIR images) without requiring advanced acquisition protocols or complex post-processing pipelines, making it suitable for routine clinical settings and retrospective analyses. Nevertheless, the sqMRI presents inherent limitations. As a semi-quantitative and observer-dependent method, it may be influenced by rater subjectivity and provides a low-resolution characterization of lesion extent and location compared to fully quantitative approaches. Techniques such as volumetric MRI offer higher spatial resolution and more precise quantification of tissue integrity, potentially allowing for a more detailed investigation of structure–function relationships. Therefore, the associations observed in the present study should be interpreted within the methodological constraints of the sqMRI approach. Future studies integrating quantitative neuroimaging techniques may further refine the understanding of the relationship between structural brain damage and variability in response to AOT. Importantly, given the observational nature of the present analyses, the reported findings should be interpreted as associations rather than evidence of causal relationships between brain lesion characteristics and response to AOT.

With respect to clinical outcome assessment, although the AHA demonstrates excellent interrater reliability, with Intraclass Correlation Coefficients (ICCs) consistently reported at 0.97 to 0.98, several methodological precautions were implemented to mitigate the potential risk of rater-related bias, including standardized video-recorded assessments scored by certified raters blinded to group allocation and time point. Moreover, the scores were carried out by two AHA certified rater therapists following a blind assessment on the same videos independently, and in case of disagreement they discussed the reasons for the scores to reach a high agreement. Nevertheless, some degree of subjectivity inherent to observational scales such as the AHA cannot be completely excluded.

Therefore, future studies with larger cohorts, quantitative measures of brain structure and function and longitudinal MRI data collection are warranted to better understand how structural and functional brain features relate with motor improvements over time. Addressing these limitations will be essential for optimizing the clinical application of AOT and advancing personalized rehabilitation in UCP.

## Data Availability

Data and all the materials are available in anonymous form upon reasonable request by contacting the corresponding author (LB) at the following link: https://doi.org/10.5281/zenodo.20159268.

## References

[ref1] Kim J. Y. Kim J. M. Ko E. Y. (2014). The effect of the action observation physical training on the upper extremity function in children with cerebral palsy. J Exerc Rehabil. 10, 176–183. doi: 10.12965/jer.140114, 25061598 PMC4106773

[ref2] Abdelhaleem N. Taher S. Mahmoud M. Hendawy A. Hamed M. Mortada H. . (2021). Effect of action observation therapy on motor function in children with cerebral palsy: a systematic review of randomized controlled trials with meta-analysis. Clin. Rehabil. 35, 51–63. doi: 10.1177/0269215520954345, 32907374

[ref3] Alamer A. Melese H. Adugna B. (2020). Effectiveness of action observation training on upper limb motor function in children with hemiplegic cerebral palsy: a systematic review of randomized controlled trials. Pediatr Health Med Ther. 11, 335–346. doi: 10.2147/PHMT.S266720, 32982541 PMC7501989

[ref4] Anderson V. Spencer-Smith M. Wood A. (2011). Do children really recover better? Neurobehavioural plasticity after early brain insult. Brain 134, 2197–2221. doi: 10.1093/brain/awr103, 21784775

[ref5] Bax M. Goldstein M. Rosenbaum P. Leviton A. Paneth N. Dan B. . (2005). Proposed definition and classification of cerebral palsy, April 2005. Dev. Med. Child Neurol. 47, 571–576. doi: 10.1017/S001216220500112X, 16108461

[ref6] Beani E. Barzacchi V. Scaffei E. Ceragioli B. Festante F. Filogna S. . (2024). Neuroanatomical correlates of gross manual dexterity in children with unilateral spastic cerebral palsy. Front. Hum. Neurosci. 18:1370561. doi: 10.3389/fnhum.2024.1370561, 38655371 PMC11035821

[ref7] Beani E. Menici V. Sicola E. Ferrari A. Feys H. Klingels K. . (2023). Effectiveness of the home-based training program tele-UPCAT (tele-monitored UPper limb children action observation training) in unilateral cerebral palsy: a randomized controlled trial. Eur J Phys Rehabil Med [Internet]. 59, 554–563. doi: 10.23736/S1973-9087.23.07945-5, 37462399 PMC10664769

[ref8] Bonini L. (2017). The extended Mirror neuron network: anatomy, origin, and functions. Neuroscientist 23, 56–67. doi: 10.1177/1073858415626400, 26747293

[ref9] Borges L. R. Fernandes A. B. Oliveira Dos Passos J. Rego I. A. O. Campos T. F. (2022). Action observation for upper limb rehabilitation after stroke. Cochrane Database Syst Rev 2022:CD011887. doi: 10.1002/14651858.CD011887.pub3, 35930301 PMC9354942

[ref10] Buccino G. Arisi D. Gough P. Aprile D. Ferri C. Serotti L. . (2012). Improving upper limb motor functions through action observation treatment: a pilot study in children with cerebral palsy. Dev. Med. Child Neurol. 54, 822–828. doi: 10.1111/j.1469-8749.2012.04334.x, 22765352

[ref11] Buccino G. Molinaro A. Ambrosi C. Arisi D. Mascaro L. Pinardi C. . (2018). Action observation treatment improves upper limb motor functions in children with cerebral palsy: a combined clinical and brain imaging study. Neural Plast. 2018, 1–11. doi: 10.1155/2018/4843985, 30123250 PMC6079352

[ref12] Buccino G. Solodkin A. Small S. L. (2006). Functions of the mirror neuron system: implications for neurorehabilitation. Cogn. Behav. Neurol. 19, 55–63. doi: 10.1097/00146965-200603000-00007, 16633020

[ref13] Buchignani B. Beani E. Pomeroy V. Iacono O. Sicola E. Perazza S. . (2019). Action observation training for rehabilitation in brain injuries: a systematic review and meta-analysis. BMC Neurol. 19:344. doi: 10.1186/s12883-019-1533-x, 31881854 PMC6935205

[ref14] Burzi V. Tealdi G. Boyd R. N. Guzzetta A. (2016). Action observation in infancy: implications for neuro-rehabilitation. Dev. Med. Child Neurol. 58, 74–77. doi: 10.1111/dmcn.13048, 27027611

[ref15] Caldú X. Reid L. B. Pannek K. Fripp J. Ballester-Plané J. Leiva D. . (2024). Tractography of sensorimotor pathways in dyskinetic cerebral palsy: association with motor function. Ann. Clin. Transl. Neurol. 11, 2609–2622. doi: 10.1002/acn3.52174, 39257055 PMC11514975

[ref16] Errante A. Fogassi L. (2020). Activation of cerebellum and basal ganglia during the observation and execution of manipulative actions. Sci. Rep. 10:12008. doi: 10.1038/s41598-020-68928-w, 32686738 PMC7371896

[ref17] Errante A. Gerbella M. Mingolla G. P. Fogassi L. (2023). Activation of cerebellum, basal ganglia and thalamus during observation and execution of mouth, hand, and foot actions. Brain Topogr. 36, 476–499. doi: 10.1007/s10548-023-00960-1, 37133782 PMC10293454

[ref18] Eyre J. (2007). Corticospinal tract development and its plasticity after perinatal injury. Neurosci. Biobehav. Rev. 31, 1136–1149. doi: 10.1016/j.neubiorev.2007.05.011, 18053875

[ref19] Fiori S. Cioni G. Klingels K. Ortibus E. Van Gestel L. Rose S. . (2014). Reliability of a novel, semi-quantitative scale for classification of structural brain magnetic resonance imaging in children with cerebral palsy. Dev. Med. Child Neurol. 56, 839–845. doi: 10.1111/dmcn.12457, 24750109

[ref20] Fiori S. Guzzetta A. Pannek K. Ware R. S. Rossi G. Klingels K. . (2015). Validity of semi-quantitative scale for brain MRI in unilateral cerebral palsy due to periventricular white matter lesions: relationship with hand sensorimotor function and structural connectivity. NeuroImage Clin. 8, 104–109. doi: 10.1016/j.nicl.2015.04.005, 26106533 PMC4473818

[ref21] Friel K. M. Kuo H. C. Carmel J. B. Rowny S. B. Gordon A. M. (2014). Improvements in hand function after intensive bimanual training are not associated with corticospinal tract dysgenesis in children with unilateral cerebral palsy. Exp. Brain Res. 232, 2001–2009. doi: 10.1007/s00221-014-3889-x, 24623352 PMC4037561

[ref22] Hari R. Kujala M. V. (2009). Brain basis of human social interaction: from concepts to brain imaging. Physiol. Rev. 89, 453–479. doi: 10.1152/physrev.00041.2007, 19342612

[ref23] Himmelmann K. Horber V. De La Cruz J. Horridge K. Mejaski-Bosnjak V. Hollody K. . (2017). MRI classification system (MRICS) for children with cerebral palsy: development, reliability, and recommendations. Dev. Med. Child Neurol. 59, 57–64. doi: 10.1111/dmcn.13166, 27325153

[ref24] Hoon A. H. Jr. Stashinko E. E. Nagae L. M. Lin D. D. Keller J. Bastian A. . (2009). Sensory and motor deficits in children with cerebral palsy born preterm correlate with diffusion tensor imaging abnormalities in thalamocortical pathways. Dev. Med. Child Neurol. 51, 697–704. doi: 10.1111/j.1469-8749.2009.03306.x, 19416315 PMC2908264

[ref25] Islam M. Nordstrand L. Holmström L. Kits A. Forssberg H. Eliasson A. (2014). Is outcome of constraint-induced movement therapy in unilateral cerebral palsy dependent on corticomotor projection pattern and brain lesion characteristics? Dev. Med. Child Neurol. 56, 252–258. doi: 10.1111/dmcn.12353, 24341408

[ref26] Kim D. H. (2020). Comparison of short- and long-time action observation training (AOT) on upper limb function in children with cerebral palsy. Physiother Pract Res. 41, 53–58. doi: 10.3233/PPR-190145

[ref27] Kirkpatrick E. Pearse J. James P. Basu A. (2016). Effect of parent-delivered action observation therapy on upper limb function in unilateral cerebral palsy: a randomized controlled trial. Dev. Med. Child Neurol. 58, 1049–1056. doi: 10.1111/dmcn.13109, 27038153

[ref28] Krumlinde-sundholm L. Eliasson A. Christin L. (2003). Development of the assisting hand assessment: a Rasch-built measure intended for children with unilateral upper limb impairments. Scand. J. Occup. Ther. 10, 16–26. doi: 10.1080/11038120310004529

[ref29] Krumlinde-Sundholm L. Holmefur M. Kottorp A. Eliasson A. C. (2007). The assisting hand assessment: current evidence of validity, reliability, and responsiveness to change. Dev. Med. Child Neurol. 49, 259–264. doi: 10.1111/j.1469-8749.2007.00259.x, 17376135

[ref30] Kuhnke N. Juenger H. Walther M. Berweck S. Mall V. Staudt M. (2008). Do patients with congenital hemiparesis and ipsilateral corticospinal projections respond differently to constraint-induced movement therapy? Dev. Med. Child Neurol. 50, 898–903. doi: 10.1111/j.1469-8749.2008.03119.x, 18811703

[ref31] Laporta-Hoyos O. Ballester-Plané J. Vázquez E. Delgado I. Narberhaus A. Póo P. . (2014). PS-247 association of motor function with basal ganglia and thalamus volumes in Dyskinetic cerebral palsy. Arch. Dis. Child. 99, A202.1–A2A202. doi: 10.1136/archdischild-2014-307384.546, 38354671

[ref32] Jeong Y. A. Lee B. H. (2020). Effect of action observation training on spasticity, Gross motor function, and balance in children with Diplegia cerebral palsy. Children 7:64. doi: 10.3390/children7060064, 32570855 PMC7346110

[ref33] Lee J. D. Park H. J. Park E. S. Oh M. K. Park B. Rha D. W. . (2011). Motor pathway injury in patients with periventricular leucomalacia and spastic diplegia. Brain 134, 1199–1210. doi: 10.1093/brain/awr021, 21385750

[ref34] Liang K. J. Chen H. L. Shieh J. Y. Wang T. N. (2021). Measurement properties of the box and block test in children with unilateral cerebral palsy. Sci. Rep. 11:20955. doi: 10.1038/s41598-021-00379-3, 34697312 PMC8545961

[ref35] Louwers A. Beelen A. Holmefur M. Krumlinde-Sundholm L. (2016). Development of the assisting hand assessment for adolescents (ad‐AHA) and validation of theAHAfrom 18 months to 18 years 58, 1303–1309. doi: 10.1111/dmcn.13168, 27291981

[ref36] Mathiowetz V. Federman S. Wiemer D. (1985). Box and block test of manual dexterity: norms for 6–19 year olds. Can. J. Occup. Ther. 52, 241–245. doi: 10.1177/000841748505200505

[ref37] Mathiowetz V. Volland G. Kashman N. Weber K. (1985). Adult norms for the box and block test of manual dexterity. Am. J. Occup. Ther. 39, 386–391. doi: 10.5014/ajot.39.6.386, 3160243

[ref38] Mu X. Nie B. Wang H. Duan S. Zhang Z. Dai G. . (2014). Spatial patterns of whole brain Grey and white matter injury in patients with occult spastic Diplegic cerebral palsy. PLoS One 9:e100451. 24964139 10.1371/journal.pone.0100451 PMC4070986

[ref39] Novak I. Morgan C. Fahey M. Finch-Edmondson M. Galea C. Hines A. . (2020). State of the evidence traffic lights 2019: systematic review of interventions for preventing and treating children with cerebral palsy. Curr. Neurol. Neurosci. Rep. 20:3. doi: 10.1007/s11910-020-1022-z, 32086598 PMC7035308

[ref40] Nuara A. Avanzini P. Rizzolatti G. Fabbri-Destro M. (2019). Efficacy of a home-based platform for child-to-child interaction on hand motor function in unilateral cerebral palsy. Dev. Med. Child Neurol. 61, 1314–1322. doi: 10.1111/dmcn.14262, 31115046

[ref41] Quadrelli E. Anzani A. Ferri M. Bolognini N. Maravita A. Zambonin F. . (2019). Electrophysiological correlates of action observation treatment in children with cerebral palsy: a pilot study. Dev. Neurobiol. 79, 934–948. doi: 10.1002/dneu.22734, 31981294

[ref42] Rizzolatti G. Craighero L. (2004). The mirror-neuron system. Annu. Rev. Neurosci. 27, 169–192. doi: 10.1146/annurev.neuro.27.070203.144230, 15217330

[ref43] Sakzewski L. Ziviani J. Boyd R. (2009). Systematic review and Meta-analysis of therapeutic Management of Upper-Limb Dysfunction in children with congenital hemiplegia. Pediatrics 123, e1111–e1122. doi: 10.1542/peds.2008-3335, 19451190

[ref44] Sanvido L. Krishnan P. Domi T. Walker K. Fehlings D. Robertson A. . (2022). Abstract TP172: clinical and neuroimaging predictors of Dyskinetic cerebral palsy. Stroke 53:172. doi: 10.1161/str.53.suppl_1.TP172

[ref45] Schertz M. Shiran S. I. Myers V. Weinstein M. Fattal-Valevski A. Artzi M. . (2016). Imaging predictors of improvement from a motor learning–based intervention for children with unilateral cerebral palsy. Neurorehabil. Neural Repair 30, 647–660. doi: 10.1177/1545968315613446, 26564999

[ref46] Sgandurra G. Biagi L. Fogassi L. Ferrari A. Sicola E. Guzzetta A. . (2020). Reorganization of action observation and sensory-motor networks after action observation therapy in children with congenital hemiplegia: a pilot study. Dev. Neurobiol. 80, 351–360. doi: 10.1002/dneu.22783, 32986904

[ref47] Sgandurra G. Biagi L. Fogassi L. Sicola E. Ferrari A. Guzzetta A. . (2018). Reorganization of the action observation network and sensory-motor system in children with unilateral cerebral palsy: an fMRI study. Neural Plast. 2018, 1–15. doi: 10.1155/2018/6950547, 30147718 PMC6083552

[ref48] Sgandurra G. Cecchi F. Beani E. Mannari I. Maselli M. Falotico F. P. . (2021). Tele-UPCAT: study protocol of a randomised controlled trial of a home-based tele-monitored UPper limb children action observation training for participants with unilateral cerebral palsy. BMJ Open 8:e017819. doi: 10.1136/bmjopen-2017-017819, 29764869 PMC5961615

[ref49] Sgandurra G. Ferrari A. Cossu G. Guzzetta A. Fogassi L. Cioni G. (2013). Randomized trial of observation and execution of upper extremity actions versus action alone in children with unilateral cerebral palsy. Neurorehabil. Neural Repair 27, 808–815. doi: 10.1177/1545968313497101, 23886886

[ref50] Simon-Martinez C. Mailleux L. Hoskens J. Ortibus E. Jaspers E. Wenderoth N. . (2025). Randomized controlled trial combining constraint-induced movement therapy and action-observation training in unilateral cerebral palsy: clinical effects and influencing factors of treatment response. Ther. Adv. Neurol. Disord. 10.1177/1756286419898065 PMC6977217 32031542

[ref51] Simon-Martinez C. Mailleux L. Ortibus E. Fehrenbach A. Sgandurra G. Cioni G. . (2018). Combining constraint-induced movement therapy and action-observation training in children with unilateral cerebral palsy: a randomized controlled trial. BMC Pediatr. 18:250. doi: 10.1186/s12887-018-1228-2, 30064396 PMC6069849

[ref52] Staudt M. Gerloff C. Grodd W. Holthausen H. Niemann G. Krägeloh-Mann I. (2004). Reorganization in congenital hemiparesis acquired at different gestational ages. Ann. Neurol. 56, 854–863. doi: 10.1002/ana.20297, 15562409

[ref53] Weinstein M. Green D. Geva R. Schertz M. Fattal-Valevski A. Artzi M. . (2014). Interhemispheric and intrahemispheric connectivity and manual skills in children with unilateral cerebral palsy. Brain Struct. Funct. 219, 1025–1040. doi: 10.1007/s00429-013-0551-5, 23571779

[ref54] Yoshida S. Hayakawa K. Yamamoto A. Okano S. Kanda T. Yamori Y. . (2010). Quantitative diffusion tensor tractography of the motor and sensory tract in children with cerebral palsy. Dev. Med. Child Neurol. 52, 935–940. doi: 10.1111/j.1469-8749.2010.03669.x, 20412261

