# Supplementary material for: Structural brain lesions and action observation therapy outcomes in unilateral cerebral palsy: an exploratory study
Source: Front Syst Neurosci. 2026 May 22;20:1766684. doi: 10.3389/fnsys.2026.1766684 (PMC13236681; doi:10.3389/fnsys.2026.1766684)
Supplement: Supplementary file 1 [file Data_Sheet_1.docx]

Supplementary Material

# Supplementary Data

| **Table S1.** sqMRI scores distribution in the overall sample (n=16) | | |
| --- | --- | --- |
|  | (mean (SD)) | (median [IQR]) |
| Ipsilesional Frontal | 1.66 (0.79) | 1.50 [1.00, 2.12] |
| Ipsilesional Temporal | 1.53 (1.18) | 1.50 [0.38, 2.62] |
| Ipsilesional Parietal | 2.28 (0.89) | 2.50 [2.00, 3.00] |
| Ipsilesiona Occipital | 0.69 (0.87) | 0.00 [0.00, 1.50] |
| Ipsilesional periventricular | 2.28 (1.17) | 2.25 [1.50, 3.50] |
| Ipsilesional middle | 2.34 (1.01) | 2.50 [1.50, 3.12] |
| Ipsilesional cortico/subcortical | 1.56 (1.44) | 1.75 [0.00, 3.00] |
| Ipsilesional Caudate | 0.00 (0.00) | 0.00 [0.00, 0.00] |
| Ipsilesional_PLIC | 0.81 (0.40) | 1.00 [1.00, 1.00] |
| Ipsilesional_Lenticular | 0.25 (0.45) | 0.00 [0.00, 0.25] |
| Ipsilesional_Thalamus | 0.69 (0.48) | 1.00 [0.00, 1.00] |
| Ipsilesional_Brainstem | 0.69 (0.48) | 1.00 [0.00, 1.00] |
| Corpus Callosum | 1.88 (0.89) | 2.00 [1.00, 2.25] |
| Contralesional Frontal | 0.06 (0.25) | 0.00 [0.00, 0.00] |
| Contralesional Temporal | 0.06 (0.25) | 0.00 [0.00, 0.00] |
| Contralesional Parietal | 0.16 (0.51) | 0.00 [0.00, 0.00] |
| Contralesiona Occipital | 0.06 (0.25) | 0.00 [0.00, 0.00] |
| Contralesional periventricular | 0.16 (0.62) | 0.00 [0.00, 0.00] |
| Contralesional middle | 0.16 (0.62) | 0.00 [0.00, 0.00] |
| Contralesional cortico/subcortical | 0.03 (0.12) | 0.00 [0.00, 0.00] |
| Contralesional Caudate | 0.00 (0.00) | 0.00 [0.00, 0.00] |
| Contralesional_PLIC | 0.00 (0.00) | 0.00 [0.00, 0.00] |
| Contralesional_Lenticular | 0.00 (0.00) | 0.00 [0.00, 0.00] |
| Contralesional_Thalamus | 0.00 (0.00) | 0.00 [0.00, 0.00] |
| Contralesional_Brainstem | 0.00 (0.00) | 0.00 [0.00, 0.00] |
| Cerebellum | 0.06 (0.25) | 0.00 [0.00, 0.00] |

| **Table S2.**  Mixed linear models, with fixed effects: time (T1, T2, and T3) adjusting by Age, Sex and **MRICS categories**, carried out on BBT_dom, BBT_non_dom, and *AHA scores* | | | | |
| --- | --- | --- | --- | --- |
| **Outcome: BBT_DOM** | | | | |
| Predictors | Coefficient | Std_Error | T_Value | P_Value |
| (Intercept) | 33.690 | 7.842 | 4.296 | 0.001 |
| TIMET1 | 4.284 | 1.690 | 2.534 | **0.015** |
| TIMET2 | 5.611 | 1.701 | 3.298 | **0.002** |
| TIMET3 | 6.005 | 1.761 | 3.411 | **0.001** |
| Age | 2.069 | 0.636 | 3.252 | **0.005** |
| SexMale | 3.184 | 4.592 | 0.693 | 0.503 |
| **MRICS_Type_III** | -5.220 | 4.904 | -1.064 | 0.311 |
| **Outcome: BBT_NON_DOM** | | | | |
| Predictors | Coefficient | Std_Error | T_Value | P_Value |
| (Intercept) | 18.004 | 10.500 | 1.715 | 0.104 |
| TIMET1 | 1.940 | 1.058 | 1.834 | 0.075 |
| TIMET2 | 2.989 | 1.076 | 2.779 | **0.008** |
| TIMET3 | 1.739 | 1.191 | 1.461 | 0.151 |
| Age | 1.072 | 0.718 | 1.492 | 0.143 |
| SexMale | -0.370 | 7.240 | -0.051 | 0.960 |
| **MRICS_Type_III** | -9.687 | 7.704 | -1.257 | 0.235 |
| **Outcome: AHA** | | | | |
| Predictors | Coefficient | Std_Error | T_Value | P_Value |
| (Intercept) | 49.194 | 10.276 | 4.787 | 0.000 |
| TIMET1 | 2.901 | 0.871 | 3.333 | **0.002** |
| TIMET2 | 2.302 | 0.907 | 2.537 | **0.015** |
| TIMET3 | 1.597 | 1.018 | 1.569 | 0.124 |
| Age | 0.410 | 0.649 | 0.632 | 0.530 |
| SexMale | 0.476 | 7.422 | 0.064 | 0.950 |
| **MRICS_Type_III** | -6.009 | 7.889 | -0.762 | 0.462 |

Models summarized in Table S2 were performed separately for each outcome (dependent variables: bbt_DOM, BBT_non_dom, and AHA scores), adjusted for covariates (TIME, age, and sex), and MRICS categories (MRICS_Type_III vs. MRICS_Type_II). Categories MRICS_Type_I, MRICS_Type_IV, and MRICS_Type_V were excluded due to insufficient data representation. Statistical significance was assessed across models with a threshold set at <0.05.
